# Chitosan as possible inhibitory agents and delivery systems in leukemia

**DOI:** 10.1186/s12935-021-02243-w

**Published:** 2021-10-18

**Authors:** Parinaz Zivarpour, Jamal Hallajzadeh, Zatollah Asemi, Fatemeh Sadoughi, Mehran Sharifi

**Affiliations:** 1 Department of Biological Sciences, Faculty of Basic Sciences, Higher Education Institute of Rab-Rashid, Tabriz, Iran; 2 Department of Biochemistry and Nutrition, Research Center for Evidence-Based Health Management, Maragheh University of Medical Sciences, Maragheh, Iran; 3 Research Center for Biochemistry and Nutrition in Metabolic Diseases, Institute for Basic Sciences, Kashan University of Medical Sciences, Kashan, Iran; 4 Department of Internal Medicine, School of Medicine, Cancer Prevention Research Center, Seyyed Al-Shohada Hospital, Isfahan University of Medical Sciences, Isfahan, Iran

**Keywords:** Leukemia, Acute leukemia, Chronic leukemia, Chitosan, Chitin

## Abstract

Leukemia is a lethal cancer in which white blood cells undergo proliferation and immature white blood cells are seen in the bloodstream. Without diagnosis and management in early stages, this type of cancer can be fatal. Changes in protooncogenic genes and microRNA genes are the most important factors involved in development of leukemia. At present, leukemia risk factors are not accurately identified, but some studies have pointed out factors that predispose to leukemia. Studies show that in the absence of genetic risk factors, leukemia can be prevented by reducing the exposure to risk factors of leukemia, including smoking, exposure to benzene compounds and high-dose radioactive or ionizing radiation. One of the most important treatments for leukemia is chemotherapy which has devastating side effects. Chemotherapy and medications used during treatment do not have a specific effect and destroy healthy cells besides leukemia cells. Despite the suppressing effect of chemotherapy against leukemia, patients undergoing chemotherapy have poor quality of life. So today, researchers are focusing on finding more safe and effective natural compounds and treatments for cancer, especially leukemia. Chitosan is a valuable natural compound that is biocompatible and non-toxic to healthy cells. Anticancer, antibacterial, antifungal and antioxidant effects are examples of chitosan biopolymer properties. The US Food and Drug Administration has approved the use of this compound in medical treatments and the pharmaceutical industry. In this article, we take a look at the latest advances in the use of chitosan in the treatment and improvement of leukemia.

## Introduction

Leukemia is a type of blood cancer that is caused by abnormal function of blood tissue [1, 2]. Leukemia manifests itself in the form of abnormal growth of immature white blood cells. This disease can kill a person if it is not diagnosed and controlled in its early stages [3, 4]. Leukemia usually manifests as high, rapid, and uncontrolled proliferation of leukocytes and their precursor cells, leading to the accumulation of immature leukocytes in the bloodstream [5, 6]. Excessive production of immature white blood cells and their entry into the bloodstream can cause anemia in patients. In 2016, statistics showed that 34,090 men and 26,050 women were suffering from leukemia and 24,400 out of these 60,140 individuals died [7]. Changes in protooncogenic genes and microRNA genes are among the most important factors involved in the development of leukemia [3, 8–10]. Leukemia can generally be divided into four types: (a) chronic lymphocytic leukemia (CLL), (b) acute lymphocytic leukemia (ALL), (c) chronic myelogenous leukemia (CML), and (d) acute myeloid leukemia (AML). While in CLL, B lymphocytes proliferate irreguralely, immature B or T lymphocytes are involved in the pathogensis of ALL. Furthermore, granulocyte precursors and immature myeloid cells are involved cells in CML and in AML, respectively [1, 11, 12]. The incidence and survival rate of different types of this heterogenous cancer are not similar: “Among adults (20 years of age and older), the most common types of leukemia are CLL (38%) and AML (31%), whereas ALL is most common in children and adolescents (ages 0 to 19 years), accounting for 74% of cases” [13]. “AML is the most common form of acute leukemia in adults and has the shortest survival (5-year survival = 24%)”. CML is also more common in adults and “It accounts for approximately 15% of newly diagnosed cases of leukemia in adults” [14–16].

The incidence of leukemia in developing countries is much higher than in other countries where urbanization problems, efforts to control infections, and high tobacco use are among the predisposing factors for leukemia in these countries [5, 17]. Patients with leukemia generally have low hemoglobin levels and the number of leukocytes in their bloodstream is high. Also, the number of platelets in these patients decreases to less than normal. Weakness, persistent paleness and yellowing of the skin, anemia, purpura, retinal hemorrhage, and lymphadenopathy are among the main symptoms that leukemia patients suffer from [5, 18].

Studies show that mutations and the lack of regulated cell death (RCD) proteins cause resistance to conventional therapies in patients suffering from leukemia. Chemotherapy and the use of corticosteroid drugs, P13K/mTOR inhibitors, tyrosine kinase inhibitors and stem cell transplantation are examples of first-line leukemia treatment methods. These treatments are invasive therapies and destroy the immune system [3, 19–21]. After chemotherapy, which is common in the treatment of almost all types of leukemia, the survival rate is less than 70% in children and less than 20% in adults [22]. In addition to chemo- and radio-therapy, allogeneic bone marrow transplantation is another option on the table for leukemia treatment, especially in adults. It seems that using stem cell transplantation is more common in AML and CML compared to ALL [23]. Thus, in recent years, efforts have been made to find treatments that show less side effects on other parts of the body and provide better quality of life similar to studies about other types of cancer [24].

Since ancient times, the use of natural ingredients to treat diseases such as cancer has been common and has helped scientists to discover and develop more effective drugs in this field [25, 26]. Epigallocatechin gallate, curcumin, quercetin, silymarin and stilbene resveratrol are examples of plant compounds that have anti-cancer potential by regulating the cell cycle and controlling the apoptosis pathway associated with p53, or have similar performance to potent chemotherapy drugs [25, 27–34].

Chitosan is also a natural compound that is non-toxic and biocompatible [35, 36]. Chitosan is a biological and cationic polysaccharide found in the skeleton of arthropods, the skin of a variety of crabs, the cell wall of fungi and the skin of insects [37–42]. *O*-carboxymethyl chitosan, *N*carboxymethyl chitosan and *N*, *O*-carboxymethyl chitosan are three types of chitosan derivatives that are produced by its carboxymethylation [38, 41]. Studies show that *O*-carboxymethyl chitosan is more compatible with blood, so it is widely used in medicine [38, 43]. Various studies have shown that chitosan has antibacterial, anti-tumor, and antioxidant effects and has significant pharmacological effects [1, 44–47]. The inhibitory effects of chitosan on degranulation and cytokine production in rats’ basophilic leukemia cells have been confirmed [1, 48]. Antioxidant activity against superoxide anion is one of the most important properties of chitosan. The antioxidant activity of chitosan is important for the production of broad-spectrum drugs with high antioxidant activity [38, 49]. Reports indicate that chitosan induces apoptosis, but chitosan carboxymethyl has the property of inhibiting apoptosis [50, 51]. Chitosan is also used in the treatment of cancer as a low-toxicity chemotherapeutic drug [38, 41]. The US Food and Drug Administration (FDA) has approved the use of chitosan in medicine and the manufacture of drugs [35, 52].

Overall, the advantages of chitosan as a drug or a delivery system has guided us through precisely looking into the studies using chitosan on one of the most lethal cancers: leukemia.

## Leukemia: risk factors and primary prevention

So far, the risk factors of leukemia have been identified in several studies. In general, the risk factors involved in developing leukemia can be divided into four categories: (a) genetic, (b) environmental, (c) familial, and (d) lifestyle factors. Studies show that in the absence of genetic risk factors, leukemia can be prevented by reducing the exposure to some risk factors, including smoking, exposure to benzene compounds and high-dose radioactive or ionizing radiation [53].

Here is a summary of some of the important factors that contribute to leukemia.

### Genetic and familial risk factors

Genetic factors only play a role in some cases of leukemia [53–55]. So far, several examples of chromosomal abnormalities leading to leukemia have been identified. Philadelphia chromosomal abnormalities play a role in the development of CML, which usually involves gene displacement between chromosomes 9 and 22 [53, 55]. Some genetic syndromes are caused by chromosomal mutations. Diseases such as Down syndrome, Bloom syndrome, ataxia telangiectasia, and Fanconi anemia can be predisposing factors for leukemia [53, 56–73]. Gene transfer between chromosomes 8 and 21 or between 12 and 21 chromosomes usually supports AML [74, 75].

Large-scale genome-wide association studies have revealed that various genomic loci with common polymorphisms are correlated with the susceptibility to ALL. Most of these polymorphisms are located in genes related to hematopoietic transcription factors, such as ERG, ARID5B, IKZF3, IKZF1, CEBPE, and GATA3. Individually, these risk alleles contribute to limited significance in clinic. However, their aggregation causes a ninefold rise in the risk of leukemia in cases with multiple risk alleles compared with cases with no risk alleles [76]. Based on the investigations done on pediatric populations, it is found that some genetic syndromes are associated with the increased risk of ALL, including Bloom syndrome, Nijmegen breakdown syndrome, Fanconi anemia, Down syndrome, and ataxia telangiectasia. Although chromosomal changes are not enough for the development of leukemia, some aberrations are characteristic of ALL, such as MLL rearrangement, t(9;22) [*BCR-ABL1*], t(12;21) [*ETV6-RUNX1*], and t(1;19) [*TCF3-PBX1*] [77].

### Environmental risk factors

#### Benzene and benzene compounds

Benzene is widely used as an important solvent in various industries for the production of materials and compounds, including printing, leather and petrochemical industries [53, 78]. Benzene compounds can affect people by smoking or in the workplace [53, 79–82]. Statistics show that mortality has increased in patients with leukemia, especially AML [53, 81, 83, 84]. From time immemorial, there is strong evidence that people with leukemia are affected by benzene compounds, which can be considered a high-risk carcinogen [81, 82, 85–93].

#### Ionizing radiation

Reports indicate that ionizing radiation is a major risk factor for AML, ALL, and CML [53]. Studies show that these rays do not play a role in CLL. Pierce et al. studied the lifespan of atomic bomb survivors from 1950 to 1990. Statistics from their study show that out of 86,572 people studied, 249 died of leukemia due to exposure to ionizing radiation [53]. A study by Preston et al. Found that about 50% of leukemia patients were exposed to ionizing radiation in 1945 in Hiroshima and Nagasaki [94].

Another source of radiation which can lead to secondary leukemia is using radiotherapy for treating other cancers. A study on patients with gynecologic malignancies treated in the past 20 years found that there is only 0.38% chance of leukemia development in these patients. However, this percent is not specific to radiotherapy and it involves leukemias developed secondary to chemotherapy, as well [95]. Another study examined patients with invasive tumors of the vulva, cervix, uterus, anus, and rectosigmoid treated with radiotherapy and found that not only the risk of developing leukemia is 72% higher in these patients, but also “the risk of secondary leukemia peaks at 5 to 10 years after primary treatment and remains elevated even 10 to 15 years after initial treatment” [96]. After all, it can be concluded that radiotherapy might have a role in initiating leukemia in patients who suffer from other types of cancer.

### Lifestyle risk factors

Obesity is a lifestyle-related risk factor for leukemia. Impaired immune response and decreased leptin levels in the blood may be functional mediators of obesity-induced leukemia [97–101]. Leptin increases the proliferation of CD4^+^ T cells and stimulates the proliferation of myelocytic and primary progenitor cells [102–104]. Some studies have shown that a daily diet rich in vegetables can reduce the risk of AML [105, 106].

Another factor in the lifestyle-related risk factors is smoking. Various epidemiological studies have confirmed the risk of leukemia, especially myeloid leukemia, in smokers [107–115]. Sandler et al. [116] showed that there was a link between smoking and acute leukemia [116]. In 2004, the International Agency for Research and Cancer and General Surgeon Carmona and colleagues found that smoking is a major risk factor for AML [53, 117–119]. Kasim et al. [54] in their case study in Canada, confirmed the significant effect of lifestyle factors on leukemia [54]. Some studies have also cited smoking as a risk factor for ALL and CLL [53, 120, 121].

## Chitosan: biochemical structure and medicinal, therapeutic properties

**Fig. 1:**
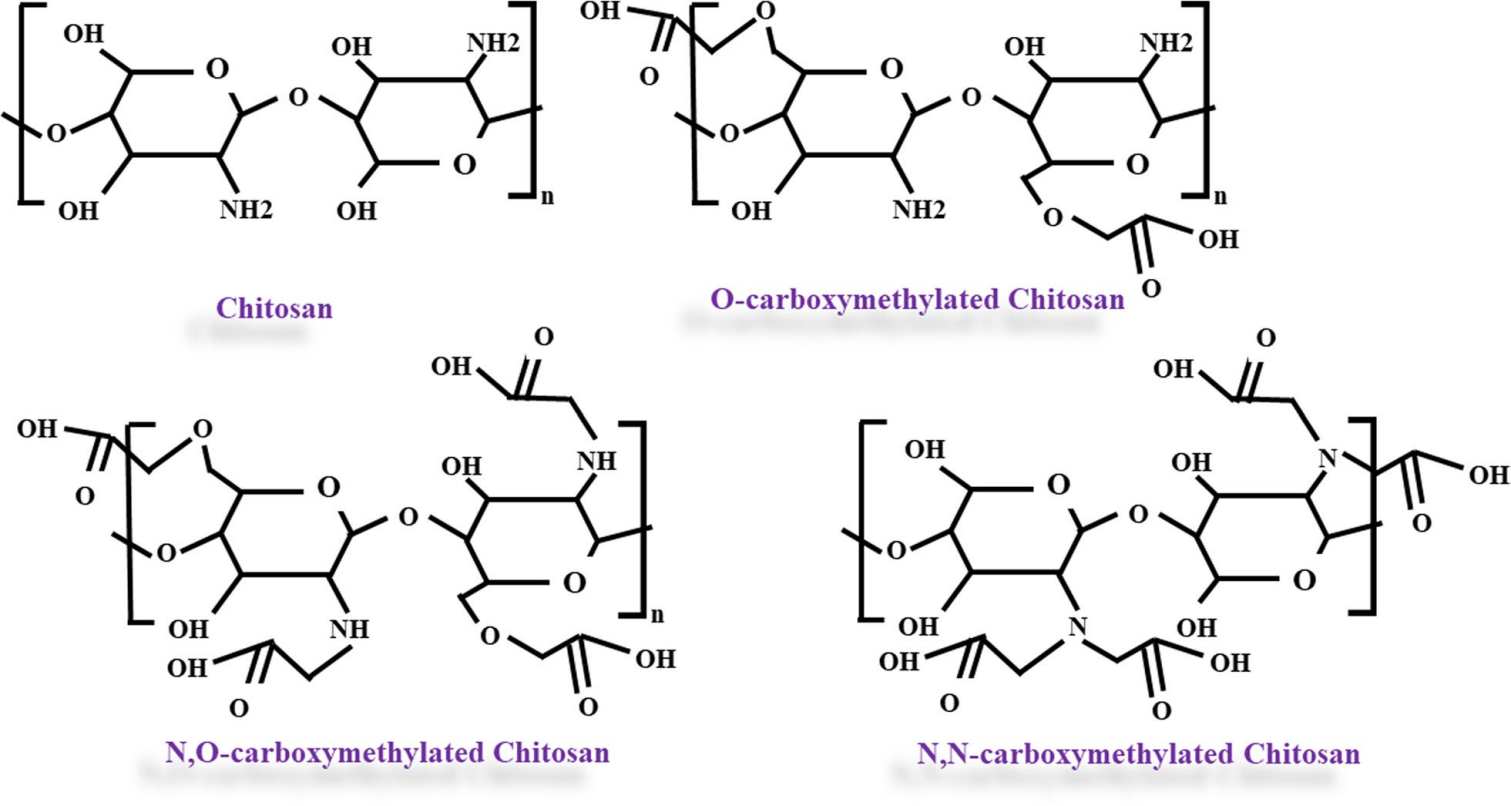
Chitosan and some of its derivatives

Chitosan is a natural polysaccharide biopolymer composed of *N*-acetyl glucosamine and glucosamine components and is obtained in industry by hydrolysing amino acetyl groups in chitin (β-(1-4)-poly-*N*-acetyl-d-glucosamine which is shown in (Fig. 1) [25, 122–127]. After cellulose, chitosan is the second most abundant substance in nature. Crabs and shrimp are the most important sources of chitosan. Chitosan is also found in the cell walls of fungi and insect scales [25, 122, 125–129]. It has many valuable properties in addition to being natural such as non-toxicity, degradability, biocompatibility, low immunogenicity, and high affinity for metals, proteins, and dyes as well as high water resistance and ductility in various forms such as gel, nanoparticles and grains. All of these valuable properties have led to chitosan being regarded as a promising effective biopolymer in the pharmaceutical industry, especially anticancer drugs. It is also used in drug delivery systems to control the process of drug release in the body [25, 123, 130–137]. The mentioned effect of chitosan is mostly relying on its effects on paracellular and transcellular transportation. Through facilitating these ways of transport and opening the tight junctions of epithelial cells, chitosan is able to ferry drugs [138]. However, how it delivers drugs to specific sites is not completely understood. It seems that its pH-dependent drug release, protein adsorption onto nanoparticles following the serum exposure, getting involved in phagocytic pathways, and the enhanced permeation and retention (EPR) effect might be the reasons why chitosan acts specifically on cancer cells [138–140].

Low chitosan solubility in physiological pH (> 6.0) is one of the factors that limit its use. Due to the carboxymethylation of chitosan, a compound called carboxymethyl chitosan is produced, which, in addition to having some of chitosan’s properties, it also has better flexibility and good solubility in water [123, 141–145]. On the other hand, the end-carboxymethyl groups in its structure are the arm that binds to drugs such as 6-mercaptopurine [123, 146, 147]. The hydrolysed compounds derived from chitosan are soluble in water. d-Glucosamine oligomer, chitosan oligosaccharide, is one of these derivatives that has antibacterial, antioxidant and anti-tumor properties and can be used in drug delivery systems [1, 44–47, 148]. Chitosan oligosaccharide can suppress degranulation and cytokine production in live mice’s basophilic leukemia cells [48]. Reports indicate that the secretion and expression of tumor necrosis factor (TNF)-α, inflammatory cytokines, and interleukin (IL)-6 are inhibited in human astrocytoma cells treated with soluble chitosan [149].

## The latest methods of using chitosan as suppressive compounds and delivery systems in leukemia patients

A combination of chemotherapy and radiotherapy is the most common treatment for various types of cancer, such as leukemia. These therapies and the drugs used in them are considered dangerous because they do not have sufficient specificity to affect the target or the desired tissue. Despite the possible cure for cancer, quality of life is deteriorating in patients who have received these treatments. Therefore, the discovery and presentation of treatment options that are selective and at the same time have fewer side effects is a necessity. Studies show that chitosan, as a small transfer biomolecule, is very valuable in the production of nano-anti-cancer drugs. In addition to its anti-cancer properties, chitosan in these drugs selectively transports small particles and drug molecules into target cellular organelles. The transfer of small drug molecules into cellular organelles increases the toxicity of the drug to cancer cells and reduces it in healthy cells. Chitosan also enhances the effects of other chemotherapy drugs combined with it [150–152]. In the following, we will discuss the latest leukemia treatment strategies that have used chitosan (Table 1).

**Table 1:** Recent experimental studies have shown the use of chitosan in the treatment and inhibition of leukemia

Form of chitosan	Concentration/nanoparticle diameter	Model	Cell line	Findings	Refs.
FA-CMCS NPs	143.9 ± 3.9 nm	In vitro	Human promyelocytic leukemia cells (HL60)	Combining folic acid modified carboxymethyl chitosan nanoparticles with Methotrexate causes toxicity to leukemia cells without affecting healthy cells	[153]
Fe3O4-PEG-LAC-chitosan-PEI NPs	≤ 100 nM	In vitro	Human chronic myelogenous leukemia cells (K562)	Combining Fe3O4-PEG-LAC-chitosan-PEI nanoparticles with survivin siRNA inhibits survivin expression and prevents leukemia progression	[158]
*S*-Nitroso-MSA-chitosan NPs	50 mmo/l	In vitro	Human chronic myelogenous leukemia cells (K562)	S-nitroso-MSA-CS nanoparticles have toxic effects on HepG2 and K562 cells without affecting healthy cells	[174]
Chitosan	60 ug/ml	In vitro	Acute lymphoblastic leukemic cells	Chitosan alters the morphology of leukemia cells and stimulates cell death	[240]
FA-CS-PTX-SPION	10–60 µM/90 ± 15 nm	In vitro	Human chronic myelogenous leukemia cells (K562)	FA-CS-PTX-SPION targets leukemia cells and directs them to apoptosis without affecting other normal cells	[186]
Chitosan NPs	≤ 100 nm	In vitro	Human T lymphocyte acute T cell leukemia BCL2 (AAA)	Chitosan nanoparticles stimulate apoptosis by inducing oxidative stress by reducing glutathione and increasing ROS in cancer cells, including leukemia without affecting healthy cells	[150]
CH-Au NPs	3.7±0.6 nm	In vitro	T-acute lymphocytic leukemia cells (CEM) and chronic myeloid leukemia cells (K562)	Treatment leukemia cells with CH-AuNPs greatly increases the production of ROS and damages mitochondria and cell nuclei	[20]
Zn-CS NPs	160.7 nm	In vitro	Human acute T-lymphocyte leukemia cells (6T-CEM)	Zinc released from Zn-CS NPs activates the first apoptotic signal Fas/CD95 and expresses apoptotic regulatory genes, leading leukemia cells to cell death	[3]
CS-AQ NPs	197 ± 16.8 nm	In vitro	Human promyelocytic leukemia cells (HL60)	CS-AQ NPs by inhibiting cell cycle in the pre-G0 phase, stimulate apoptosis in leukemia cells	[25]
Fe3O4-CMC Genistein NPs	7–14 nm	In vitro	Acute leukemia lymphoma cells (ALL)	The gradual secretion of genistein from Fe3O4-CMC Genistein NPs, suppresses the growth of leukemia cells and stimulates apoptosis in them for a long time, and SPIONs and carboxymethylated chitosan enhance this genistein function	[38]
Ag NPs-Chitosan	20–40 nm	In vivo (mouse model)	Acute myeloid leukemia cells	Chitosan enhances the antioxidant and anti-cancer potentials of Ag NPs	[230]

### Folic acid modified carboxymethyl chitosan (fa-cmcs) self-assemble nanoparticles: toxicity to leukemia cells

Carboxymethyl chitosan (CMCS) is one of the water-soluble derivatives of chitosan that is used in different pharmaceutical industries. Combining CMCS with folic acid (FA) forms a hydrophobic compound consisting of pteroyl, in which case in the aquatic environment the amphiphilic groups easily and spontaneously form nanoparticles. In this case, CMCS combined with folic acid (FA) is an effective nanocarrier for long-term drug release. The desired hydrophobic drug accumulates in the hydrophobic microdomains of these nanoparticles and the drug is gradually released through the polysaccharide skeleton into the target tissue [153–157]. HU and colleagues show that folic acid modified carboxymethyl chitosan nanoparticles can act as a highly efficient drug carrier and gradually release drug particles into the target tissue or cell [153]. In their study, HU and colleagues placed Methotrexate in Folic Acid Modified Carboxymethyl Chitosan nanoparticles and tested its effect on human promyelocytic leukemia cells (HL60) in vitro. They showed that these nanoparticles could specifically transport the drug to target cells without affecting healthy cells and gradually release it by changing the pH in the target environment [153]. Direct administration of anticancer drugs, due to the lack of adequate specificity for tumor cells, also affects other healthy cells and causes side effects. Therefore, the design of particles that can direct these drugs specifically to tumor cells will lead to a safer and more effective treatment because in this case, the toxicity of the drug to normal cells is reduced and its toxicity to cancer cells is increased.

### Fe_3_O_4_-PEG-LAC-chitosan-PEI nanoparticles: survivin siRNA delivery

In recent years, the use of RNA interference in gene therapy methods has been widely welcomed by researchers. However, one of the major problems with this treatment is the transfer of small interfering RNAs (siRNAs) to target tumor cells [158–164]. Various pathways are involved in the inhibition or progression of cancer in the body, the most important of which are cell death and survival. Anti-apoptotic genes that are directly linked to caspases encode factors that suppress apoptotic processes by suppressing the apoptotic proteins. The Survivin gene is involved in a variety of biological processes, including cell cycle regulation, cell protection, and cell death suppression, and through these processes, it maintains the survival of cancer cells. This gene is highly expressed in cancer cells. Reports indicate that if survivin expression is suppressed, cancer cells become sensitive to anticancer compounds and drugs [158, 165, 166]. From these findings, it can be concluded that inhibiting Survivin expression is a potentially valuable target for cancer treatment and suppression of its progression.

Polyethyleneglycol (PEG) and chitosan are two compounds which are commonly used in the synthesis of oligonuleotide cationic particles [167–172]. Polyethyleneimine (PEI) is one of the most effective carriers of the gene, with its “proton-sponge effect” in laboratory and in vivo. Polyethyleneimine acts as a buffer around the endosome, releasing compounds into the cytoplasm [163]. Chitosan polysaccharide effectively coats the nanoparticles and thereby, stabilizes them and prevents particles from accumulating. In the synthesis of these carrier nanoparticles, PEG reduces PEI toxicity and ensures the stability of colloidal particles. On the other hand, PEG, in addition to its high biocompatibility, prevents the deposition of nanoparticles [158, 173]. Arami et al. [158] designed the “Fe3O4-PEG-LAC-chitosan-PEI” nanoparticle carrier, which due to the properties of the compounds used in it, has a sufficient positive charge to react with siRNAs [158]. In their study, they used the nanoparticle to transfer survivin siRNA to human breast cancer cells (MCF-7) and human chronic myelogenous leukemia cells (K562) in vitro. Their findings show that Fe3O4-PEG-LAC-chitosan-PEI nanoparticles combine well with survivin siRNA, and their nanoscale-size makes them a good carrier for gene delivery in the treatment of various cancers such as breast and leukemia. Survivin siRNA therapy using Fe3O4-PEG-LAC-chitosan-PEI nanoparticle is a safe and specific treatment that does not affect healthy cells [158]. Therefore, this nanoparticle is a valuable case study for the treatment of cancers based on siRNA delivery.

### *S*-Nitroso-MSA-chitosan nanoparticles: toxicity to leukemia cells

The free radical nitric oxide (NO) produced in the body is involved in regulating important processes such as wound healing, cellular communication, dilation of blood vessels, prevention of platelet aggregation, immune defense, and bronchial dilation [174–184]. Inside the body, nitric oxide is produced by the oxidation of l-arginine to l-citrulline by the activity of nitric oxide synthase enzyme (NOS) [179]. Studies show that NO plays an important role in the defense of the immune system and has anti-tumor properties [174, 184, 185]. Therefore, the use of NO-releasing nanoparticles in cancer treatment is a new treatment strategy that requires several studies to further evaluate its side effects and benefits. Pelegrino et al. [174] used chitosan to design a NO-releasing nanoparticle that has antibacterial, antifungal, and anti-cancer effects [174]. In their study, they used chitosan to produce capsules of low molecular weight mercaptosuccinic acid and examined the effect of its toxicity on human hepatocellular carcinoma (HepG2) and human chronic myeloid leukemia cells (K562). Mercaptosusic acid contains the thiol group (*S*-nitroso-MSA), which actually acts as an NO donor. Therefore, the nanoparticle composition designed by Pelegrino et al. is “*S*-nitroso-MSA-CS”. The results obtained after treating HepG2 and K562 cells with this nanoparticle in vitro show that the gradual release of NO from *S*-nitroso-MSA-CS nanoparticles have toxic effects on HepG2 and K562 cells but no effect on healthy noncancerous cells [174]. Therefore, the use of this nanoparticle can be a promising treatment based on NO therapy in cancers such as leukemia.

### FA-CS-PTX-SPION: nanocarrier for paclitaxel drug delivery

Most common cancer treatments and medications have devastating side effects and are not specific enough for tumor cells. Today, there is a lot of research to achieve and provide new methods of treatment that, while effective, have minimal side effects. Tumor drug delivery is one of the new treatment strategies that has been welcomed by researchers in recent years. In this method, a specific drug is placed inside a biocompatible compound. This capsule-like structure targets cancer cells and gradually releases the drug into the environment [186–188]. Therefore, in these conditions, healthy non-cancerous cells are protected from destructive effects, and on the other hand, the toxicity of the drug to cancer cells increases. In fact, it is relatively safer and more effective. There have been many studies on the use of chitosan biocompatible biopolymers in drug delivery systems, and very good results have been obtained. This polysaccharide has the ability to absorb proteins and metals, and due to its adhesive properties to the mucosa, it can increase the absorption of the drug in the tissues and control its release [186, 189–192]. The role of magnetic nanoparticles, such as super-magnetic iron oxide nanoparticles (SPION), in cell isolation processes, cell apoptosis, and enzyme inactivation has been extensively investigated. The results show that SPION performs well in drug delivery systems and genes [52, 186, 193–197].

Paclitaxel (PTX) is a drug used to treat a variety of cancers, such as cervical, breast, pancreatic, lung, ovarian carcinoma, head and neck carcinoma, and acute leukemia. Lack of water solubility, low biocompatibility and resistance of cancer cells to Paclitaxel are some of the problems in the use of this drug [198–200]. So far, many efforts have been made to provide appropriate methods for using this drug, one of which is the efficient use of “FA-CS-PTX-SPION” nanoparticles, which Al-Musawi and his colleagues succeeded in designing using chitosan and SPION [186, 201–203]. By loading Paclitaxel into these nanoparticles, they were able to provide a more effective treatment for leukemia patients [186]. They concluded that FA-CS-PTX-SPION by targeting leukemia cells induced apoptosis in them and did not have a detrimental effect on normal noncancerous cells [186]. Therefore, the use of FA-CS-PTX-SPION can be considered as a new safe and effective treatment method in leukemia patients.

### Chitosan nanoparticles: ROS-dependent cell death

Reports indicate that a sudden and rapid increase in the amount of reactive oxygen species (ROS) in cancer cells makes them irreversibly vulnerable to external factors. Free oxygen radicals act as a second messenger in various signaling pathways that regulate the activity of enzymes involved in cell death and play an important role in regulating apoptosis [150, 204–206]. Sarangapani et al. [150] showed that the use of chitosan nanoparticles induces selective induction of apoptosis in leukemia cells [150]. Chitosan nanoparticles stimulate apoptosis by inducing oxidative stress by reducing glutathione and increasing ROS in cancer cells, including leukemia [150]. CH-AuNPs are another chitosan nanoparticles designed by Carolina et al. [20]. They used gold nanoparticles to design these new nanoparticles. Their findings show that treatment T-acute lymphocytic leukemia cells (CEM) and chronic myeloid leukemia cells (K562) with CH-AuNPs greatly increases the production of reactive oxygen species (ROS) and damages mitochondria and cell nuclei. They also found that CH-AuNPs induced apoptotic cell death in T-acute lymphocytic leukemia cells and induced necrotic cell death in chronic myeloid leukemia cells. These nanoparticles do not affect healthy non-cancerous cells [20]. Therefore, these nanoparticles have pro-apoptotic properties and use of them is a promising treatment for cancer cells that has no detrimental effects on healthy cells.

### Chitosan-nanoparticles-linked zinc (Zn-CS NPs): apoptosis inducer

Zinc is one of the body’s essential nutrients that has antioxidant properties and is very valuable for participating in the biosynthesis of proteins and DNA. Zinc is involved in the proper regulation of most cellular functions such as erythrocytes, bone cells, DNA replication and RNA transcription, neutrophils, interferon gamma secretion, and genetic division in the cell [3, 207]. Zinc supports the immune system’s response to a variety of chronic diseases such as cardiovascular disease, carcinomas and leukemia. Research has shown that supplements reduce the risk of leukemia. Studies have shown that serum zinc levels are lower in leukemia patients than in healthy individuals. In these patients, the risk of systemic errors in zinc metabolism is higher [3, 4, 208–210]. Saravanakumar et al. [3] used zinc and chitosan to design nanoparticles that could serve as new treatments for diseases caused by zinc deficiency and acute leukemia [3]. The results of their study show that after treating leukemia cells with Zn-CS NPs, zinc is gradually released from nanoparticles and activates the apoptosis pathway by targeting cancer cells. Zinc released from ZnCSNPs activates the first apoptotic signal Fas/CD95 and expresses the genes that regulate apoptosis, causing 70% damage to acute T-lymphocyte leukemia and eventually cell death [3].

### Chitosan coated anthraquinone nanoparticles: suppressing the cell growth

One of the most widely used anticancer drugs is the anthraquinone (AQ) group, including epirubicin, daunorubicin, mitoxantrone, and doxorubicin [25, 211]. These anti-cancer drugs prevent cancer such as leukemia from progressing by inhibiting cell growth and proliferation [25, 212]. The mechanism of anti-cancer function of anthraquinone is very complex [213, 214]. The DNA interfering agents of these drugs, by placing between two strands of DNA molecules, cause the strands to separate. DNA damage occurs due to production of free radicals especially ROS, in response to inhibition of topoisomerase II and induction of apoptosis by p53 and ROS-induced inhibition of topoisomerase II. Anthraquinone also stimulate apoptosis through mitochondrial pathways, Akt/PKB, and c-Jun N-terminal kinase [25, 215–219].

Redah and colleagues studied acute myeloid leukemia (HL-60) in a research [25]. To increase the effectiveness of anthraquinone and reduce its side effects, they designed chitosan nanoparticles, CS-AQ NPs, and loaded anthraquinone into it. Their findings show that CS-AQ nanoparticles inhibit the proliferation and growth of leukemia cells by stopping the cell cycle in the pre-G0 phase and directing them toward apoptosis [25]. To assess the severity of the nanoparticles toxicity on leukemia cells, they evaluated the amount of released lactate dehydrogenase (LDH) into the cell culture medium. The level of this enzyme increased significantly after 24 h. They found that by increasing the dose of nanoparticles, also the amount of secreted enzymes increased. Therefore, it can be said that these nanoparticles toxicity for leukemia cells depends on the prescribed dose [25]. Cellular studies in the Redah’s research confirm the DNA fragments presence after treatment of leukemia cells with CS-AQ NPs in the cell culture medium. The presence of these fragments supports the apoptosis process in leukemia cells [25]. Therefore, by inhibiting the cell cycle in the pre-G0 phase, these nanoparticles stimulate apoptosis in leukemia cells.

### Fe_3_O_4_-CMC-genistein nanoparticles: cell growth deterrence and apoptosis induction

Genistein is a soy isoflavone that has anti-cancer properties and can be used as an herbal chemotherapy drug. Genistein can induce apoptosis by inhibiting topoisomerase II and inhibit cell proliferation [220–224]. Many attempts have been made to discover a suitable method that can increase the effects of genistein. One of these methods is the use of magnetic nanoparticles with carboxymethylated chitosan (CMC) designed by Ghasemi et al. [38]. Magnetic nanoparticles, including SPIONs, are widely used in drug delivery systems due to their ease of synthesis, biocompatibility and their ability to absorb a variety of drugs [225–229]. By designing “Fe3O4-CMC-genistein” nanoparticles, Ghasemi and colleagues were able to amplify the anti-cancer effects of genistein at a lower dose in leukemia cells so that other healthy cells could be protected from its effects [38]. Their results show that the gradual secretion of genistein from these nanoparticles suppresses the growth of leukemia cells and stimulates apoptosis in them for a long time, and SPIONs and carboxymethylated chitosan enhance this genistein function [38].

### Ag NPs-chitosan: cytotoxicity effect

Metal nanoparticles are a good choice for drug delivery due to their wide surface area. In recent years, among the various types of metal nanoparticles, silver nanoparticles have received more attention due to their antimicrobial, antioxidant and non-toxic potential for healthy cells. Silver nanoparticles have also shown amazing anti-cancer effects [230–234]. Hemmati and colleagues first designed silver nanoparticles using chitosan, which have anti-cancer properties against mouse leukemia cells. The results of their study show that chitosan enhances the antioxidant and anti-cancer potentials of Ag NPs [230]. Therefore, Ag NPs-chitosan can be used as a chemotherapeutic drug in the treatment of leukemia, although the implementation of this work requires more clinical and human experiments.

## Conclusions

Due to the unsuccessful therapeutic and diagnostic procedures, leukemia is considered as a fatal disease. The conventional treatments for leukemia are invasive and toxic to the immune system. Furthermore, due to their nonspecific action, a reduction in the life-quality of patients is common. Since ancient times, the use of natural ingredients to treat diseases such as cancer has been common and has helped scientists to discover and develop more effective drugs in this field [31, 235, 236]. Chitosan is an example of these natural compounds that is non-toxic and biocompatible [31]. Due to the flexibility of chitosan, researchers use it to design and manufacture various nanoparticles for drug delivery purposes [237].

However, chitosan applications are not limited to drug delivery and it has shown a great potential for bone marrow transplant. For instance, an injectable hydrogel based on dextran and chitosan is reported to be effective for growth and differentiation of bone marrow derived mesenchymal stem cells [238]. Another study has also indicated that chitosan and collagen- based scaffolds impregnated with bone marrow mesenchymal stem cells improve neuropathological injury in rats with traumatic brain injury [239]. Currently, there is no evidence showing the effectiveness of chitosan in bone marrow or hematopoietic stem cell transplant in leukemia. However, further studies in this area may reveal new opportunities for treating leukemia patients.

According to the reviewed evidence, chitosan-based nanoparticles are great candidates for being used in the establishment of drug delivery systems for leukemia. Specifically targeting cancerous cells, increasing the efficacy of chemotherapeutic drugs, inducing apoptotic, and suppressing the growth of cancerous cells are some of the properties which makes these nanoparticles suitable as a replacement of conventional therapies. Interestingly, except for drugs, it seems that delivering siRNAs and mercaptosuccinic acid as a NO donor might also be effective for overcoming leukemia; However, these methods are not well-investigated and further evidence are required in these fields.

Overall, nanomedicine is upgrading our therapeutic approaches against cancer and thus, it is expected that in coming years, it would take the place of current risky methods. In this regard, we think that chitosan would be a great aid for facilitating this process, at least in the case of leukemia.

## Future directions

In our knowledge, chitosan applications are not limited to drug delivery and it has shown a great potential for bone marrow transplant. For instance, an injectable hydrogel based on dextran and chitosan is reported to be effective for growth and differentiation of bone marrow derived mesenchymal stem cells [238]. Another study has also indicated that chitosan and collagen- based scaffolds impregnated with bone marrow mesenchymal stem cells improve neuropathological injury in rats with traumatic brain injury [239]. Currently, there is no evidence showing the effectiveness of chitosan in bone marrow or hematopoietic stem cell transplant in leukemia. However, further studies in this area may reveal new opportunities for treating leukemia patients. Furthermore, we still have a long way since we can widely use chitosan nanostructures in clinics; however, more human studies would facilitate this way and speed up the process of finding novel therapies for treating leukemia.

## Data Availability

Not applicable.
